# Signal Peptidase Complex Subunit 1 Participates in the Assembly of Hepatitis C Virus through an Interaction with E2 and NS2

**DOI:** 10.1371/journal.ppat.1003589

**Published:** 2013-08-29

**Authors:** Ryosuke Suzuki, Mami Matsuda, Koichi Watashi, Hideki Aizaki, Yoshiharu Matsuura, Takaji Wakita, Tetsuro Suzuki

**Affiliations:** 1 Department of Virology II, National Institute of Infectious Diseases, Tokyo, Japan; 2 Research Institute for Microbial Diseases, Osaka University, Osaka, Japan; 3 Department of Infectious Diseases, Hamamatsu University School of Medicine, Shizuoka, Japan; University of California, San Diego, United States of America

## Abstract

Hepatitis C virus (HCV) nonstructural protein 2 (NS2) is a hydrophobic, transmembrane protein that is required not only for NS2-NS3 cleavage, but also for infectious virus production. To identify cellular factors that interact with NS2 and are important for HCV propagation, we screened a human liver cDNA library by split-ubiquitin membrane yeast two-hybrid assay using full-length NS2 as a bait, and identified signal peptidase complex subunit 1 (SPCS1), which is a component of the microsomal signal peptidase complex. Silencing of endogenous SPCS1 resulted in markedly reduced production of infectious HCV, whereas neither processing of structural proteins, cell entry, RNA replication, nor release of virus from the cells was impaired. Propagation of Japanese encephalitis virus was not affected by knockdown of SPCS1, suggesting that SPCS1 does not widely modulate the viral lifecycles of the *Flaviviridae* family. SPCS1 was found to interact with both NS2 and E2. A complex of NS2, E2, and SPCS1 was formed in cells as demonstrated by co-immunoprecipitation assays. Knockdown of SPCS1 impaired interaction of NS2 with E2. Our findings suggest that SPCS1 plays a key role in the formation of the membrane-associated NS2-E2 complex via its interaction with NS2 and E2, which leads to a coordinating interaction between the structural and non-structural proteins and facilitates the early step of assembly of infectious particles.

## Author Summary

Viruses hijack host cells and utilize host-derived proteins for viral propagation. In the case of hepatitis C virus (HCV), many host factors have been identified that are required for genome replication; however, only a little is known about cellular proteins that interact with HCV proteins and are important for the viral assembly process. The C-terminal half of nonstructural protein 2 (NS2), and the N-terminal third of NS3, form the NS2-3 protease that cleaves the NS2/3 junction. NS2 also plays a key role in the viral assembly process independently of the protease activity. We performed split-ubiquitin yeast two-hybrid screening and identified signal peptidase complex subunit 1 (SPCS1), which is a subunit of the microsomal signal peptidase complex. In this study, we provide evidence that SPCS1 interacts with both NS2 and E2, resulting in E2-SPCS1-NS2 complex formation, and has a critical role in the assembly of infectious HCV particles. To our knowledge, SPCS1 is the first NS2-interacting cellular factor that is involved in regulation of the HCV lifecycle.

## Introduction

Over 170 million people worldwide are chronically-infected with hepatitis C virus (HCV), and are at risk of developing chronic hepatitis, cirrhosis, and hepatocellular carcinoma [1]. HCV is an enveloped virus of the family *Flaviviridae*, and its genome is an uncapped 9.6-kb positive-strand RNA consisting of the 5′ untranslated region (UTR), an open reading frame encoding viral proteins, and the 3′ UTR [2]. A precursor polyprotein is further processed into structural proteins (Core, E1, and E2), followed by p7 and nonstructural (NS) proteins (NS2, NS3, NS4A, NS4B, NS5A, and NS5B), by cellular and viral proteases. The structural proteins (Core to E2) and p7 reside in the N-terminal region, and are processed by signal peptidase from the polyprotein. NS2, NS3, and NS4A are prerequisites for proteolytic processing of the NS proteins. NS3 to NS5B are considered to assemble into a membrane-associated HCV RNA replicase complex. NS3 also possesses activities of helicase and nucleotide triphosphatase. NS4 is a cofactor that activates the NS3 protease. NS4B induces vesicular membrane alteration. NS5A is considered to play an important but undefined role in viral RNA replication. NS5B is the RNA-dependent RNA polymerase. It is now accepted that NS proteins, such as NS2, NS3, and NS5A, contribute to the assembly or release of infectious HCV [3]–[9].

NS2 protein is a transmembrane protein of 21–23 kDa, with highly hydrophobic N-terminal residues forming transmembrane helices that insert into the endoplasmic reticulum (ER) membrane [5], [10]. The C-terminal part of NS2 resides in the cytoplasm, enabling zinc-stimulated NS2/3 autoprotease activity together with the N-terminal domain of NS3. The crystal structure of the C-terminal region of NS2 reveals a dimeric cysteine protease containing two composite active sites [11]. Prior work showed that NS2 is not essential for RNA replication of subgenomic replicons [12]; however, the protein is required for virus assembly independently of protease activity [5], [6]. Several adaptive mutations in NS2 that increase virus production have been reported [13]–[17]. In addition, there is increasing evidence for genetic and biochemical interaction of NS2 with other HCV proteins, including E1, E2, p7, NS3-4A, and NS5A [10], [18]–[25]. Thus, NS2 is now suggested to act as a scaffold to coordinate interactions between the structural and NS proteins for viral assembly. However, the molecular mechanism by which NS2 is involved in virus assembly remains unclear.

In this study, we identified signal peptidase complex subunit 1 (SPCS1) as a host factor that interacts with NS2 by yeast two-hybrid screening with a split-ubiquitin system. SPCS1 is a component of the microsomal signal peptidase complex which is responsible for the cleavage of signal peptides of many secreted or membrane-associated proteins. We show that SPCS1 is a novel host factor that participates in the assembly process of HCV through an interaction with NS2 and E2.

## Results

### SPCS1 is a novel host protein that interacts with HCV NS2 protein

To gain a better understanding of the functional role of NS2 in the HCV lifecycle, we screened a human liver cDNA library by employing a split-ubiquitin membrane yeast two-hybrid system with the use of NS2 as a bait. It is known that the split ubiquitin-based two-hybrid system makes it possible to study protein-protein interactions between integral membrane proteins at the natural sites of interactions in cells [26]. From the screening, several positive clones were identified from the 13 million transformants, and the nucleotide sequences of the clones were determined. A BLAST search revealed that one of the positive clones encodes a full-length coding region of signal peptidase complex subunit 1 (SPCS1). SPCS1 is a component of the microsomal signal peptidase complex which consists of five different subunit proteins in mammalian cells [27]. Although catalytic activity for SPCS1 has not been indicated to date, a yeast homolog of this subunit is involved in efficient membrane protein processing as a component of the signal peptidase complex [28].

**Figure 1:**
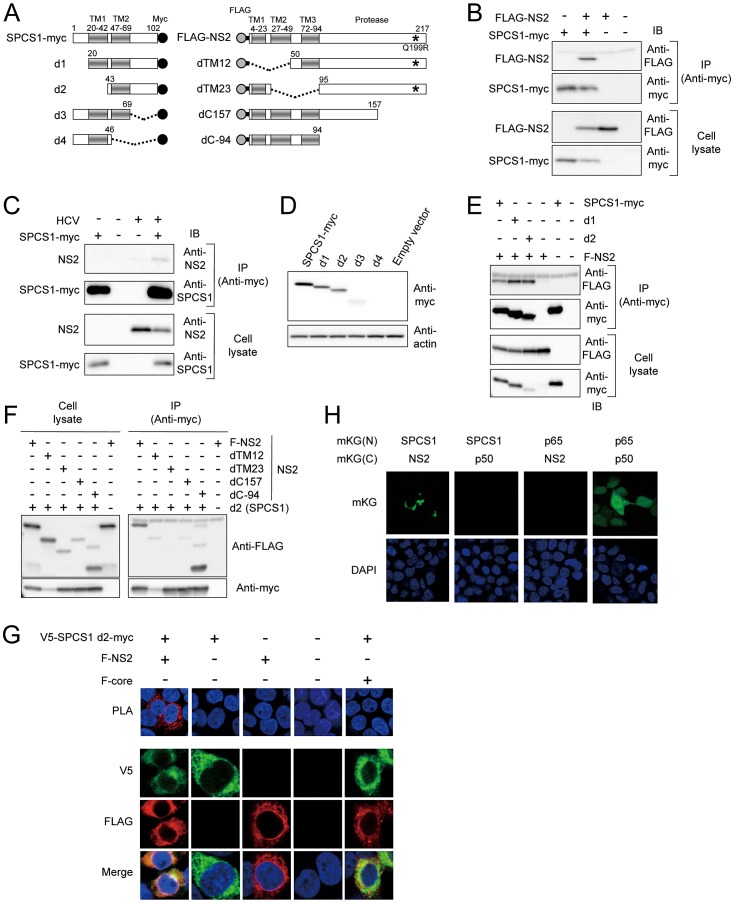
Interaction of HCV NS2 protein with SPCS1 in mammalian cells. (A) Expression constructs of SPCS1-myc and FLAG-NS2 used in this study. TM regions are represented as gray. Myc-tag regions are depicted by the black circles. Gray circles and bold lines indicated FLAG-tag and spacer (GGGGS) sequences, respectively. Adaptive mutations are indicated as asterisks. Positions of the aa resides are indicated above the boxes. (B) 293T cells were co-transfected with a FLAG-tagged NS2 expression plasmid in the presence of a SPCS1-myc expression plasmid. Cell lysates of the transfected cells were immunoprecipitated with anti-myc antibody. The resulting precipitates and whole cell lysates used in immunoprecipitation (IP) were examined by immunoblotting using anti-FLAG- or anti-myc antibody. An empty plasmid was used as a negative control. (C) HCVcc infected Huh-7 cells were transfected with a SPCS1-myc expression plasmid. Cell lysates of the transfected cells were immunoprecipitated with anti-myc antibody. The resulting precipitates and whole cell lysates used in immunoprecipitation (IP) were examined by immunoblotting using anti-NS2 or anti-SPCS1 antibody. (D) Expression of SPCS1-myc and its deletion mutants. 293T cells were transfected with indicated plasmids. The cell lysates were examined by immunoblotting using anti-myc or anti-actin antibody. (E) Cells were co-transfected with indicated plasmids, and then lysates of transfected cells were immunoprecipitated with anti-myc antibody. The resulting precipitates and whole cell lysates used in IP were examined by immunoblotting using anti-FLAG- or anti-myc antibody. (F) Lysates of the transfected cells were immunoprecipitated with anti-myc antibody. The resulting precipitates (right panel) and whole cell lysates used in IP (left panel) were examined by immunoblotting using anti-FLAG or anti-myc antibody. (G) 293T cells were transfected with indicated plasmids. 2 days posttransfection, cells were fixed and permeabilized with Triton X-100, then subjected to in situ PLA (Upper) or immunofluorescence staining (Lower) using anti-FLAG and anti-V5 antibodies. (H) Detection of the SPCS1-NS2 interaction in transfected cells using the mKG system. 293T cells were transfected by indicated pair of mKG fusion constructs. Twenty-four hours after transfection, cell were fixed and stained with DAPI, and observed under a confocal microscope.

To determine the specific interaction of NS2 with SPCS1 in mammalian cells, FLAG-tagged NS2 (FLAG-NS2; Fig. 1A) was co-expressed in 293T cells with myc-tagged SPCS1 (SPCS1-myc; Fig. 1A), followed by co-immunoprecipitation and immunoblotting. SPCS1 was shown to be co-immunoprecipitated with NS2 (Fig. 1B). Co-immunoprecipitation of SPCS1-myc with NS2 was also observed in the lysate of Huh-7 cells infected with cell culture-produced HCV (HCVcc) derived from JFH-1 isolate [29] (Fig. 1C). To determine the region of SPCS1 responsible for the interaction with NS2, deletion mutants of myc-tagged SPCS1 were constructed (Fig. 1A) and co-expressed with FLAG-tagged NS2. Since the expression of C-terminal deletion mutants, d3 and d4, was difficult to detect (Fig. 1D), N-terminal deletions (d1 and d2) as well as wild-type SPCS1 were subjected to immunoprecipitation analysis. SPCS1-myc, -d1, and -d2 were co-immunoprecipitated with NS2 (Fig. 1E), suggesting that the SPCS1 region spanning amino acids (aa) 43 to 102 is involved in its interaction with NS2. Next, to identify the NS2 region responsible for its interaction with SPCS1, deletion mutants for FLAG-NS2 (Fig. 1A) were co-expressed with SPCS1-myc-d2 in cells, followed by being immunoprecipitated with anti-myc antibody. SPCS1 was co-immunoprecipitated with the NS2 deletions, except for a mutant lacking transmembrane (TM) 2 and TM3 (dTM23) domains (Fig. 1F). These finding suggests that the TM3 region of NS2 is involved in the interaction with SPCS1.

To investigate SPCS1-NS2 interaction *in situ*, the proximity ligation assay (PLA) [30], which is based on antibodies tagged with circular DNA probes, was used. Only when the antibodies are in close proximity, the probes can be ligated together and subsequently be amplified with a polymerase. We were able to detect PLA signal predominantly in the cytoplasm of the cells expressing FLAG-NS2 and SPCS1-myc-d2 tagged with V5 at N-terminus (Fig. 1G). By contrast, the PLA signal was not observed in the context of NS2-Core co-expression. We further analyzed the SPCS1-NS2 interaction by the monomeric Kusabira-Green (mKG) system [31], which is based on fusion proteins with complementary fragments (mKG-N and mKG-C) of the monomeric coral fluorescent reporter protein. When the mKG fragments are in close proximity due to the protein-protein interaction, the mKG fragments form a beta-barrel structure and emit green fluorescence. Co-expression of SPCS1-mKG-N and NS2-mKG-C fusion proteins in cells reconstituted green cellular fluorescence as shown in Fig. 1H. Thus, these results represented structures with SPCS1 and NS2 in close proximity, and strongly suggest their physical interaction in cells.

### SPCS1 participates in the propagation of infectious HCV particles

**Figure 2:**
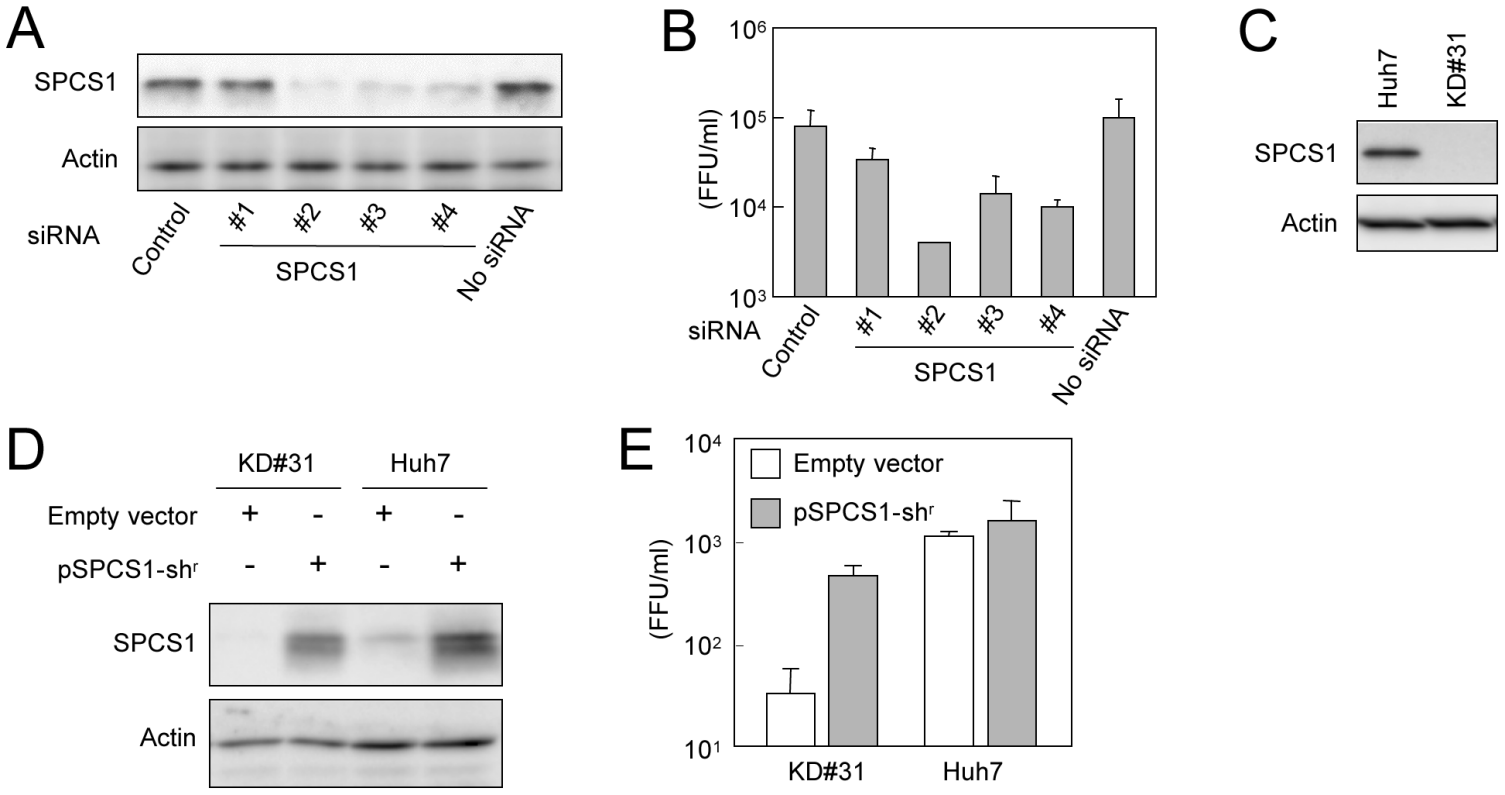
Effect of SPCS1 knockdown on the production of HCV. (A) Huh7.5.1 cells were transfected with four different siRNAs targeted for SPCS1 or control siRNA at a final concentration of 15 nM, and infected with HCVcc at a multiplicity of infection (MOI) of 0.05 at 24 h post-transfection. Expression levels of endogenous SPCS1 and actin in the cells were examined by immunoblotting using anti-SPCS1 and anti-actin antibodies at 3 days post-infection. (B) Infectious titers of HCVcc in the supernatant of cells infected as above were determined at 3 days postinfection. (C) Huh-7 cells were transfected with pSilencer-SPCS1, and hygromycin B-resistant cells were selected. The SPCS1-knockdown cell line established (KD#31) and parental Huh-7 cells were subjected to immunoblotting to confirm SPCS1 knockdown. (D) KD#31 cells or parental Huh-7 cells were transfected with RNA pol I-driven full-length HCV plasmid in the presence or absence of shRNA-resistant SPCS1 expression plasmid. Expression levels of SPCS1 and actin in the cells at 5 days post-transfection were examined by immunoblotting using anti-SPCS1 and anti-actin antibodies. (E) Infectious titers of HCVcc in the supernatants of transfected SPCS1-knockdown cells or parental Huh-7 cells at 5 days post-transfection were determined.

To investigate the role(s) of endogenous SPCS1 in the propagation of HCV, four small interfering RNAs (siRNAs) for SPCS1 with different target sequences or scrambled control siRNA were transfected into Huh7.5.1 cells, followed by infection with HCVcc. Among the four SPCS1-siRNAs, the highest knockdown level was observed by siRNA #2. siRNAs #3 and #4 showed moderate reductions of SPCS1 expression, and only a marginal effect was obtained from siRNA #1 (Fig. 2A). As indicated in Fig. 2B, the infectious viral titer in the culture supernatant was significantly reduced by the knockdown of SPCS1. It should be noted that the infectious titers correlated well with the expression levels of endogenous SPCS1. siRNA #2 reduced the HCV titer to ∼5% of the control level in Huh7.5.1 cells. To rule out the possibility of off-target effect of SPCS1-siRNA on HCV propagation, we also used “C911” mismatch control siRNAs in which bases 9 through 11 of siRNAs are replaced with their complements but other parts of antisense- and sense-strand sequences are kept intact. These mismatch designed-control siRNAs have been shown to reduce the down-regulation of the targeted mRNA, but maintains the off-target effects of the original siRNA [32]. The C911 controls against SPCS1-siRNA #2, #3, and #4 (C911-#2, -#3, and -#4) showed little effect on knockdown of SPCS1 as well as propagation of HCV (Fig. S1A and B).

We further determined the loss- and gain-of-function of SPCS1 on HCV propagation in an SPCS1-knockdown cell line. To this end, Huh-7 cells were transfected with a plasmid encoding a short hairpin RNA (shRNA) targeted to SPCS1 and were selected with hygromycin B, resulting in clone KD#31 where little or no expression of SPCS1 was detectable (Fig. 2C). KD#31 cells and parental Huh-7 cells were transfected with an RNA polymerase I (pol)-driven full-genome HCV plasmid [33] in the presence or absence of an expression plasmid for shRNA-resistant SPCS1 (SPCS1- sh^r^). Western blotting confirmed the expression levels of SPCS1 in cells (Fig. 2D). As expected, viral production in the culture supernatants of the transfected cells was significantly impaired in SPCS1-knockdown cells compared with parental Huh-7 cells (Fig. 2E white bars). Expression of SPCS1- sh^r^ in KD#31 cells recovered virus production in the supernatant to a level similar to that in the parental cells. Expression of SPCS1- sh^r^ in parental Huh-7 cells did not significantly enhance virus production. Taken together, these results demonstrate that SPCS1 has an important role in HCV propagation, and that the endogenous expression level of SPCS1 is sufficient for the efficient propagation of HCV.

**Figure 3:**
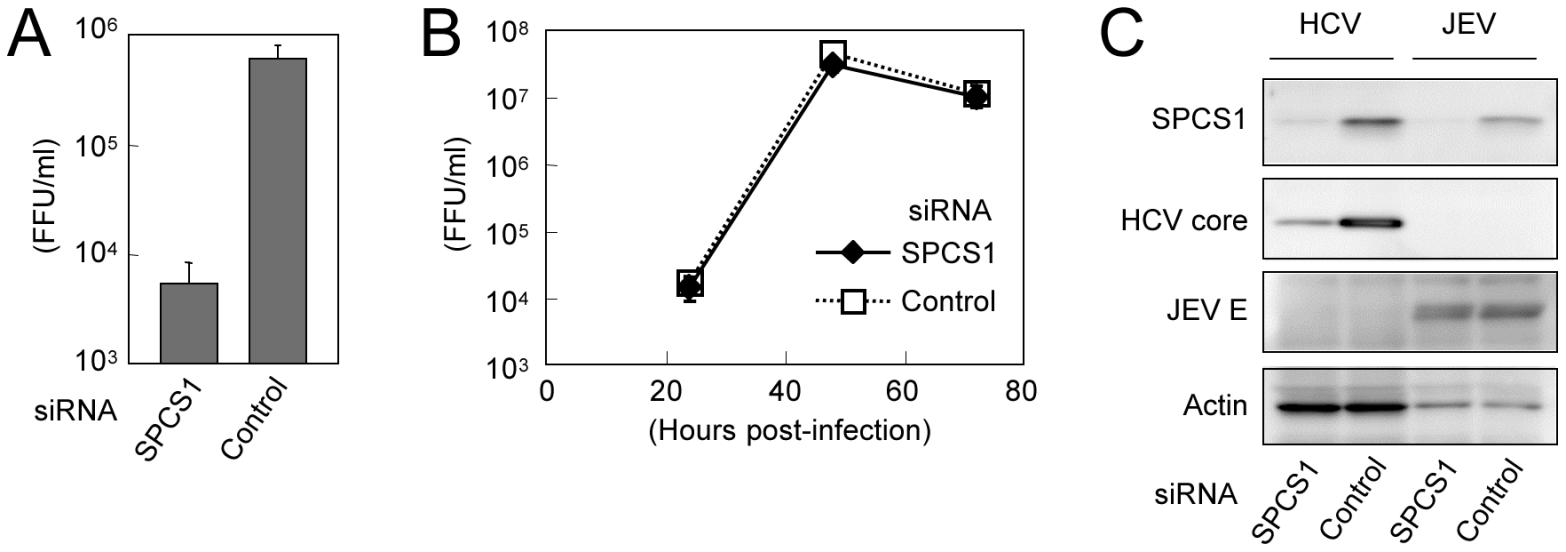
Effect of SPCS1 knockdown on the propagation of JEV.Huh7.5.1 cells were transfected with SPCS1 siRNA or control siRNA at a final concentration of 10 nM, and infected with JEV or HCVcc at an MOI of 0.05 at 24 h post-transfection. (A) Infectious titers of HCVcc in the supernatant at 3 days post-infection were determined. (B) Infectious titers of JEV in the supernatant at indicated time points were determined. (C) Expression levels of endogenous SPCS1 and actin as well as viral proteins in the cells were determined by immunoblotting using anti-SPCS1, anti-actin, anti-HCV core, and anti-JEV antibodies 3 days post-infection.

A typical feature of the *Flaviviridae* family is that their precursor polyprotein is processed into individual mature proteins mediated by host ER-resident peptidase(s) and viral-encoded protease(s). We therefore next examined the role of SPCS1 in the propagation of Japanese encephalitis virus (JEV), another member of the *Flaviviridae* family. SPCS1 siRNAs or control siRNA were transfected into Huh7.5.1 cells followed by infection with JEV or HCVcc. Although knockdown of SPCS1 severely impaired HCV production (Fig. 3A), the propagation of JEV was not affected under the SPCS1-knockdown condition (Fig. 3B). Expression of the viral proteins as well as knockdown of SPCS1 were confirmed (Fig. 3C). This suggests that SPCS1 is not a broadly active modulator of the flavivirus lifecycle, but rather is involved specifically in the production of certain virus(es) such as HCV.

### Knockdown of SPCS1 exhibits no influence on the processing of HCV proteins and the secretion of host-cell proteins

**Figure 4:**
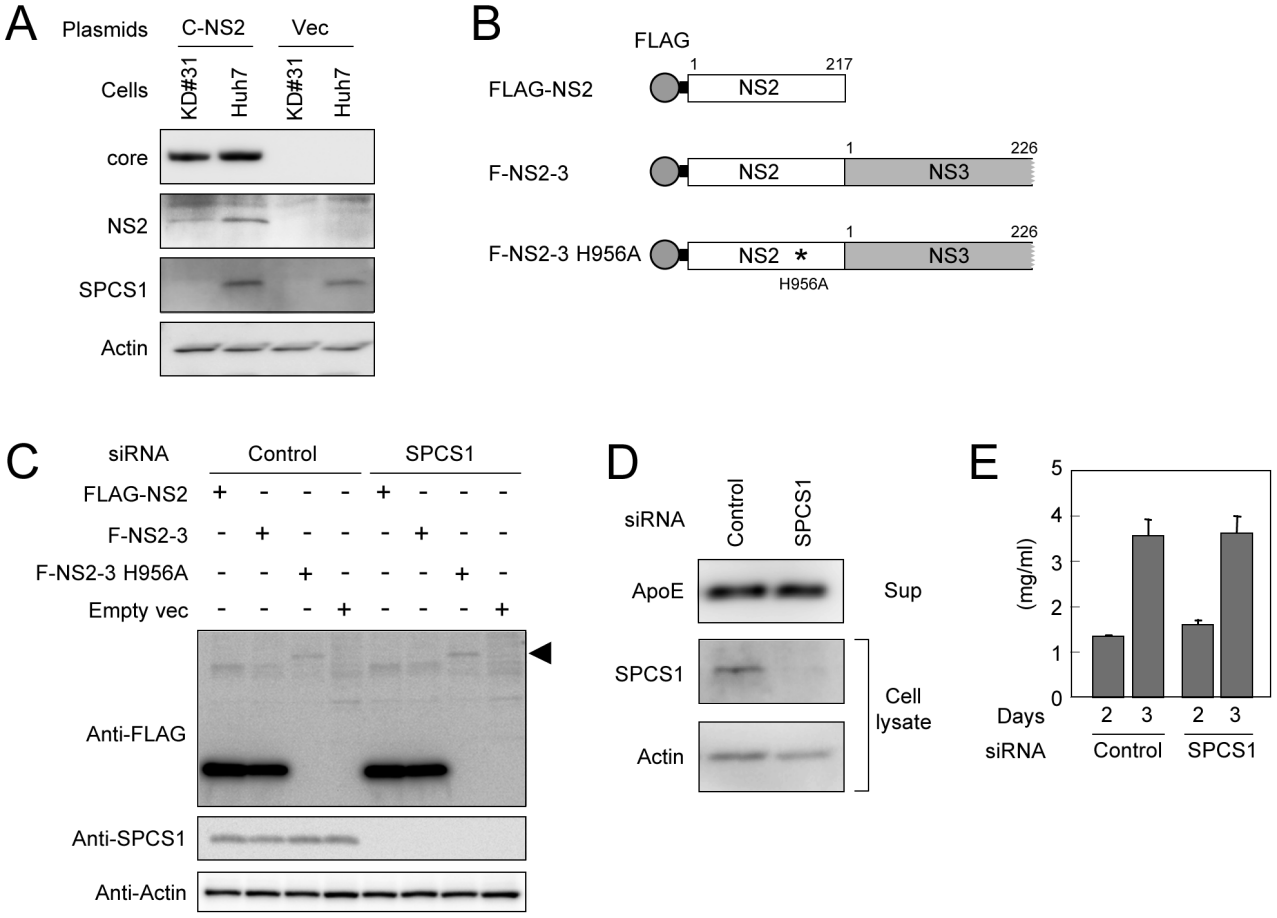
Effect of SPCS1 knockdown on the processing of HCV structural proteins and secretion of host proteins. (A) Core-NS2 polyprotein was expressed in KD#31 cells or parental Huh-7 cells. Core, NS2, SPCS1, and actin were detected by immunoblotting 2 days post-transfection. (B) Expression constructs of NS2 and NS2/3 proteins. His to Ala substitution mutation at aa 956 in NS2 is indicated by an asterisk. Gray circles and bold lines indicate FLAG-tag and the spacer sequences, respectively. Positions of the aa residues are indicated above the boxes. (C) Effect of SPCS1 knockdown on processing at the NS2/3 junction. Huh-7 cells were transfected with SPCS1 siRNA or control siRNA at a final concentration of 30 nM, and then transfected with plasmids for FLAG-NS2, F-NS2-3, or F-NS2-3 with a protease-inactive mutation (H956A). NS2 in cell lysates was detected by anti-FLAG antibody 2 days post-transfection. Arrowhead indicates unprocessed NS2-3 polyproteins. (D) Effect of SPCS1 knockdown on the secretion of apoE. Huh7.5.1 cells were transfected with SPCS1 siRNAs or control siRNA at a final concentration of 20 nM, and apoE in the supernatant and SPCS1 and actin in the cells were detected 3 days post-transfection. (E) Effect of SPCS1 knockdown on the secretion of albumin. Huh7.5.1 cells were transfected with SPCS1 siRNA or control siRNA, and albumin in the culture supernatants at 2 and 3 days post-transfection was measured by ELISA.

Since SPCS1 is a component of the signal peptidase complex, which plays a role in proteolytic processing of membrane proteins at the ER, it may be that SPCS1 is involved in processing HCV proteins via interacting with ER membranes. To address this, the effect of SPCS1 knockdown on the processing of HCV precursor polyproteins in cells transiently expressing the viral Core-NS2 region was analyzed. Western blotting indicated that properly processed core and NS2 were observed in KD#31 cells as well as Huh-7 cells (Fig. 4A). No band corresponding to the unprocessed precursor polyprotein was detected in either cell line (data not shown). We also examined the effect of SPCS1 knockdown on the cleavage of the NS2/3 junction mediated by NS2/3 protease. Processed NS2 was detected in both cell lines with and without SPCS1 knockdown, which were transfected with wild-type or protease-deficient NS2-3 expression plasmids (Fig. 4B & C).

Signal peptidase plays a key role in the initial step of the protein secretion pathway by removing the signal peptide and releasing the substrate protein from the ER membrane. It is now accepted that the secretion pathways of very-low density lipoprotein or apolipoprotein E (apoE) are involved in the formation of infectious HCV particles and their release from cells [34], [35]. ApoE is synthesized as a pre-apoE. After cleavage of its signal peptide in the ER, the protein is trafficked to the Golgi and trans-Golgi network before being transported to the plasma membrane and secreted. As shown in Fig. 4D, the secreted levels of apoE from Huh-7 cells with knocked-down of SPCS1 were comparable to those from control cells. In addition, the level of albumin, an abundant secreted protein from hepatocytes, in the culture supernatants of the cells was not influenced by SPCS1 knockdown (Fig. 4E). These data suggest that the knockdown of SPCS1 has no influence on the processing of viral and host secretory proteins by signal peptidase and HCV NS2/3 protease.

### SPCS1 is involved in the assembly process of HCV particles but not in viral entry into cells and RNA replication

**Figure 5:**
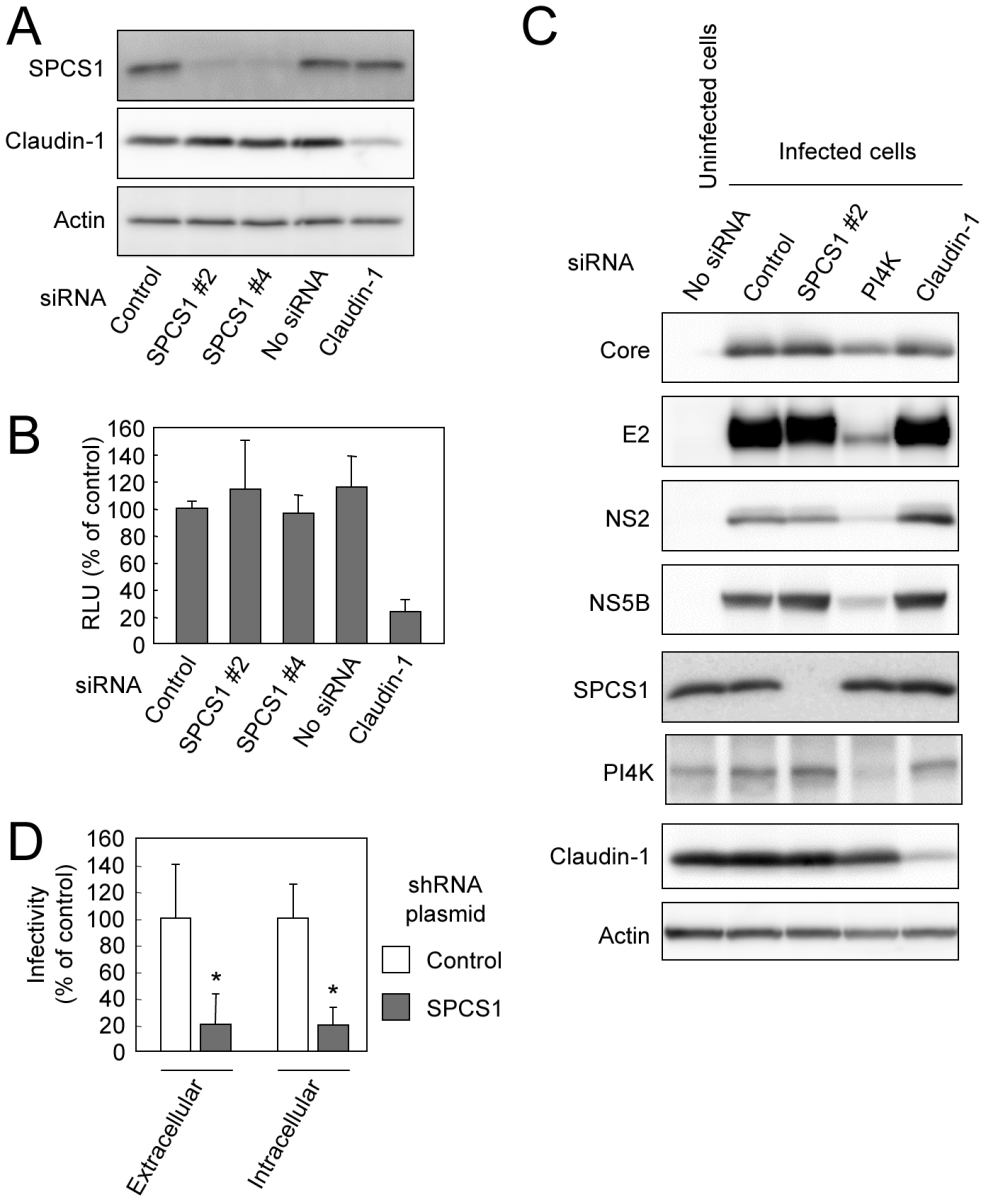
Effect of SPCS1 knockdown on entry into cells, genome replication, and assembly or release of infectious virus. (A) Huh7.5.1 cells were transfected with siRNA for SPCS1 or claudin1, or control siRNA at a final concentration of 30 nM. Expression levels of endogenous SPCS1, claudin-1, and actin in the cells at 2 days post-transfection were examined by immunoblotting using anti-SPCS1, anti-actin, and anti-claudin-1 antibodies. (B) Huh7.5.1 cells transfected with indicated siRNAs were infected with HCVtcp at 2 days post-transfection. Luciferase activity in the cells was subsequently determined at 2 days post-infection. Data are averages of triplicate values with error bars showing standard deviations. (C) Effect of SPCS1 knockdown on replication of HCV genome. HCV-infected Huh-7 cells transfected with siRNA for SPCS1, PI4K or claudin1, or control siRNA at a final concentration of 30 nM. Expression levels of HCV proteins as well as endogenous SPCS1, PI4K, claudin-1, and actin in the cells at 3 days post-transfection were examined by immunoblotting. (D) HCV infectivity in Huh7.5.1 cells inoculated with culture supernatant and cell lysate from Huh7-25 cells transfected with pSilencer-SPCS1 or control vector along with pHH/JFH1am at 5 days post-transfection. Statistical differences between Control and SPCS1 knockdown were evaluated using Student's t-test. *p<0.005 vs. Control.

To further address the molecular mechanism(s) of the HCV lifecycle mediated by SPCS1, we examined the effect of SPCS1 knockdown on viral entry and genome replication using single-round infectious trans-complemented HCV particles (HCVtcp) [33], of which the packaged genome is a subgenomic replicon containing a luciferase reporter gene. This assay system allows us to evaluate viral entry and replication without the influence of reinfection. Despite efficient knockdown of SPCS1 (Fig. 5A), luciferase activity expressed from HCVtcp in SPCS1-knockdown cells was comparable to that in control or non-siRNA-transfected cells (Fig. 5B), suggesting that SPCS1 is not involved in viral entry into cells and subgenomic RNA replication. As a positive control, knockdown of claudin-1, a cell surface protein required for HCV entry, reduced the luciferase activity. We also examined the effect of SPCS1 knockdown on full-genome replication using HCVcc-infected cells. Despite efficient knockdown of SPCS1, expression of HCV proteins was comparable to that in control cells (Fig. 5C). By contrast, knockdown of PI4 Kinase (PI4K), which is required for replication of HCV genome, led to decrease in expression of HCV proteins. As cells that had already been infected with HCV were used, knockdown of claudin-1 had no effect on HCV protein levels. These data suggest that SPCS1 is not involved in viral entry into cells and the viral genome replication. We also observed properly processed Core, E2, NS2 and NS5B in SPCS1-knockdown cells in consistent with the result as shown in Fig. 4A, indicating no effect of SPCS1 on HCV polyprotein processing.

Next, to investigate whether SPCS1 is involved in the assembly or release of infectious particles, SPCS1-shRNA plasmid along with a pol I-driven full-genome HCV plasmid [33] were transfected into CD81-negative Huh7-25 cells, which can produce infectious HCV upon introduction of the viral genome, but are not permissive to HCV infection [36]. It is therefore possible to examine viral assembly and the release process without viral reinfection. The infectivity within the transfected cells as well as supernatants was determined 5 days post-transfection. Interestingly, both intra- and extracellular viral titers were markedly reduced by SPCS1 knockdown (Fig. 5C).

Taken together, in the HCV lifecycle, SPCS1 is most likely involved in the assembly of infectious particles rather than cell entry, RNA replication, or release from cells.

### Role of SPCS1 in complex formation between NS2 and E2

**Figure 6:**
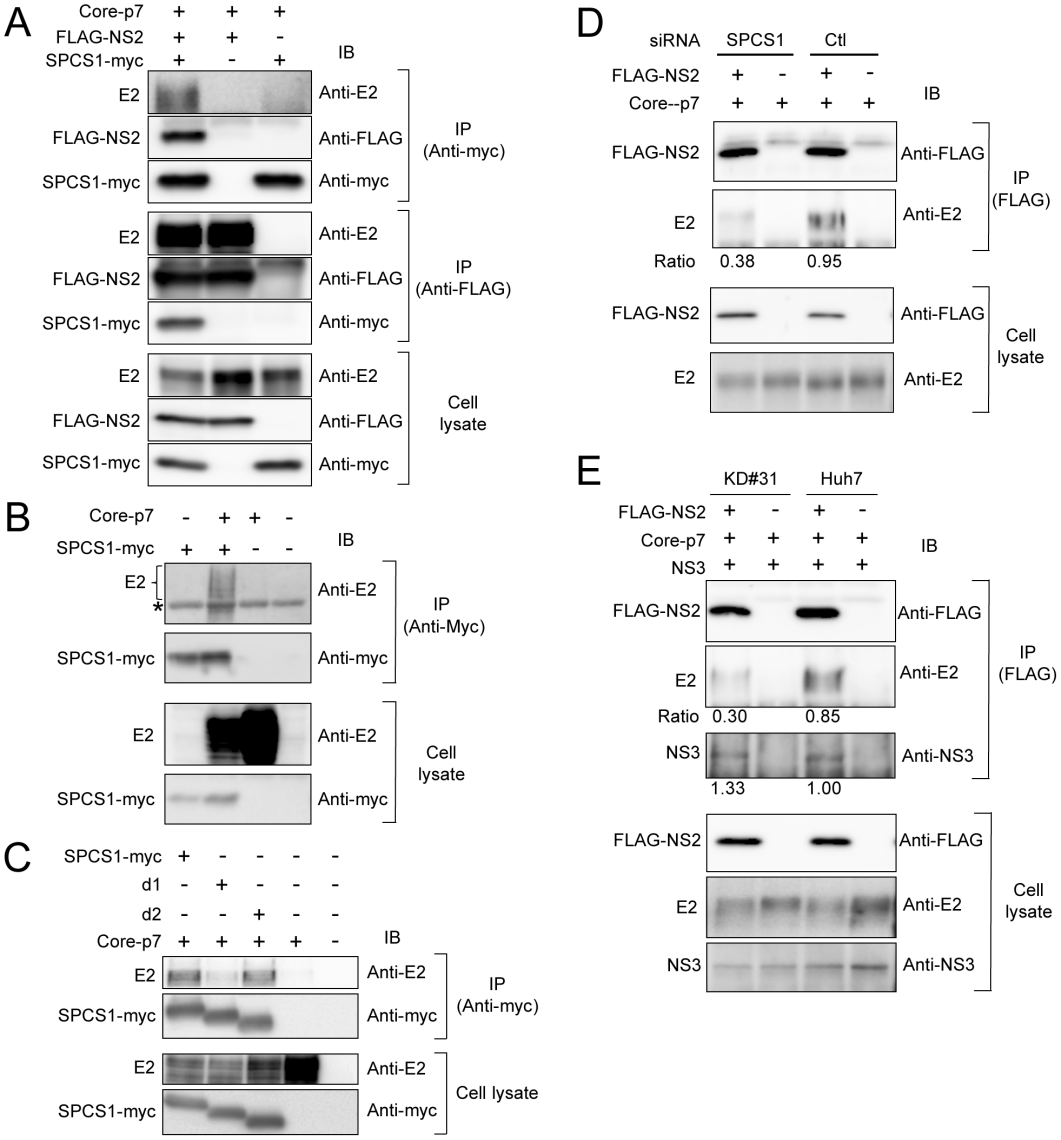
SPCS1 forms a complex with NS2 and E2. (A) Lysates of cells, which were co-transfected with Core-p7, FLAG-NS2, and SPCS1-myc expression plasmids, were immunoprecipitated with anti-myc or anti-FLAG antibody. The resulting precipitates and whole cell lysates used in IP were examined by immunoblotting using anti-E2, anti-FLAG, or anti-myc antibody. An empty plasmid was used as a negative control. (B) Cells were transfected with Core-p7 expression plasmid in the presence or absence of SPCS1-myc expression plasmid. The cell lysates of the transfected cells were immunoprecipitated with anti-myc antibody. The resulting precipitates and whole cell lysates used in IP were examined by immunoblotting using anti-E2 or anti-myc antibody. An empty plasmid was used as a negative control. The bands corresponding to immunoglobulin heavy chain are marked by an asterisk. (C) Cells were co-transfected with Core-p7 and SPCS1-myc expression plasmids. The cell lysates of the transfected cells were immunoprecipitated with anti-myc antibody. The resulting precipitates and whole cell lysates used in IP were examined by immunoblotting using anti-E2 or anti-myc antibody. (D) Huh7.5.1 cells were transfected with SPCS1 siRNA or control siRNA at a final concentration of 20 nM. After 24 h, Huh7.5.1 cells were then co-transfected with FLAG-NS2 and Core-p7 expression plasmids. The lysates of transfected cells were immunoprecipitated with anti-FLAG antibody, followed by immunoblotting with anti-FLAG and anti-E2 antibodies. Immunoblot analysis of whole cell lysates was also performed. Intensity of E2 bands was quantified, and the ratio of immunoprecipitated E2 to E2 in cell lysate was shown. Similar results were obtained in 2 independent experiments. (E) KD#31 cells and parental Huh-7 cells were co-transfected with FLAG-NS2, Core-p7, and NS3 expression plasmids. The lysates of transfected cells were immunoprecipitated with anti-FLAG antibody followed by immunoblotting with anti-FLAG, anti-E2, and anti-NS3 antibodies. Immunoblot analysis of whole cell lysates was also performed. The ratio of immunoprecipitated E2 or NS3 to E2 or NS3 in cell lysate, respectively, were shown.

It has been shown that HCV NS2 interacts with the viral structural and NS proteins in virus-producing cells [18]–[21], and that some of the interactions, especially the NS2-E2 interaction, are important for the assembly of infectious HCV particles. However, the functional role of NS2 in the HCV assembly process has not been fully elucidated. To test whether SPCS1 is involved in the interaction between NS2 and E2, cells were co-transfected with expression plasmids for E2, FLAG-NS2, and SPCS1-myc. E2 and NS2 were co-immunoprecipitated with SPCS1-myc, and E2 and SPCS1-myc were co-immunoprecipitated with FLAG-NS2 (Fig. 6A), suggesting the formation of an E2-NS2-SPCS1 complex in cells. To investigate the interaction of SPCS1 with E2 in the absence of NS2, HCV Core-p7 polyprotein or E2 protein were co-expressed with SPCS1-myc in cells, followed by immunoprecipitation with anti-myc antibody. As shown in Fig. 6B and Fig. S2, E2 was co-immunoprecipitated with SPCS1-myc. The interaction between SPCS1 and E2 was further analyzed *in situ* by PLA and mKG system. Specific signals indicating formation of the SPCS1-E2 complex were detected in both assays (Fig. S3), suggesting physical interaction between SPCS1 and E2 in cells.

We further determined the region of SPCS1 responsible for the interaction with E2 by co-immunoprecipitation assays. Full-length and deletion mutant d2 of SPCS1 (Fig. 1A) were similarly co-immunoprecipitated with E2, while only a limited amount of d1 mutant SPCS1 (Fig. 1A) was co-precipitated (Fig. 6C). It may be that the aa 43–102 region of SPCS1, which was identified as the region involved in the NS2 interaction (Fig. 1D), is important for its interaction with E2, and that deletion of the N-terminal cytoplasmic region leads to misfolding of the protein and subsequent inaccessibility to E2.

Finally, to understand the significance of SPCS1 in the NS2-E2 interaction, Huh7.5.1 cells with or without SPCS1 knockdown by siRNA were transfected with expression plasmids for Core-p7 and FLAG-NS2, followed by co-immunoprecipitation with anti-FLAG antibody. As shown in Fig. 6D, the NS2-E2 interaction was considerably impaired in the SPCS1-knockdown cells as compared to that in the control cells. A similar result was obtained in the stable SPCS1-knockdown cell line (Fig. 6E). In contrast, in that cell line, the interaction of NS2 with NS3 was not impaired by SPCS1 knockdown (Fig. 6E).

These results, together with the above findings, suggest that SPCS1 is required for or facilitates the formation of the membrane-associated NS2-E2 complex, which participates in the proper assembly of infectious particles.

## Discussion

In this study, we identified SPCS1 as a novel host factor that interacts with HCV NS2, and showed that SPCS1 participates in HCV assembly through complex formation with NS2 and E2. In general, viruses require host cell-derived factors for proceeding and regulating each step in their lifecycle. Although a number of host factors involved in genome replication and cell entry of HCV have been reported, only a few for viral assembly have been identified to date. To our knowledge, this is the first study to identify an NS2-interacting host protein that plays a role in the production of infectious HCV particles.

NS2 is a hydrophobic protein containing TM segments in the N-terminal region. The C-terminal half of NS2 and the N-terminal third of NS3 form the protease, which is a prerequisite for NS2-NS3 cleavage. In addition, it is now accepted that this protein is essential for particle production [4]–[6], [12]. However, the mechanism of how NS2 is involved in the assembly process of HCV has been unclear.

So far, two studies have screened for HCV NS2 binding proteins by yeast two-hybrid analysis [37], [38]. Erdtmann et al. reported that no specific interaction was detected by a conventional yeast hybrid screening system using full-length NS2 as a bait, probably due to hampered translocation of the bait to the nucleus [37]. They further screened a human liver cDNA library using NS2 with deletion of the N-terminal TM domain, and CIDE-B protein, a member of the CIDE family of apoptosis-inducing factors, was identified. However, whether CIDE-B is involved in the HCV lifecycle and/or viral pathogenesis is unclear. de Chassey et al. reported several cellular proteins as potential NS2 binding proteins using NS2 with N-terminal deletion as a bait [38]. Involvement of these proteins in the HCV lifecycle is also unclear. In our study, to screen for NS2-binding partners using full-length NS2 as a bait, we utilized a split-ubiquitin yeast two-hybrid system that allows for the identification of interactions between full-length integral membrane proteins or between a full-length membrane-associated protein and a soluble protein [39]. SPCS1 was identified as a positive clone of an NS2-binding protein, but proteins that have been reported to interact with NS2 were not selected from our screening.

SPCS1 is a component of the signal peptidase complex that processes membrane-associated and secreted proteins in cells. The mammalian signal peptidase complex consists of five subunits, SPCS1, SPCS2, SPCS3, SEC11A, and SEC11C [27]. Among them, the functional role of SPCS1 is still unclear, and SPCS1 is considered unlikely to function as a catalytic subunit according to membrane topology [40]. The yeast homolog of SPCS1, Spc1p, is also known to be nonessential for cell growth and enzyme activity [28], [41]. Interestingly, these findings are consistent with the results obtained in this study. Knockdown of SPCS1 did not impair processing of HCV structural proteins (Fig. 4A) or secretion of apoE and albumin (Fig. 4B and C), which are regulated by ER membrane-associated signal peptidase activity. The propagation of JEV, whose structural protein regions are cleaved by signal peptidase, was also not affected by the knockdown of SPCS1 (Fig. 3B). SPCS1, SPCS2, and SPCS3 are among the host factors that function in HCV production identified from genome-wide siRNA screening [42]. It seemed that knockdown of SPCS1 had a higher impact on the later stage of viral infection compared to either SPCS2 or SPCS3, which are possibly involved in the catalytic activity of the signal peptidase.

**Figure 7:**
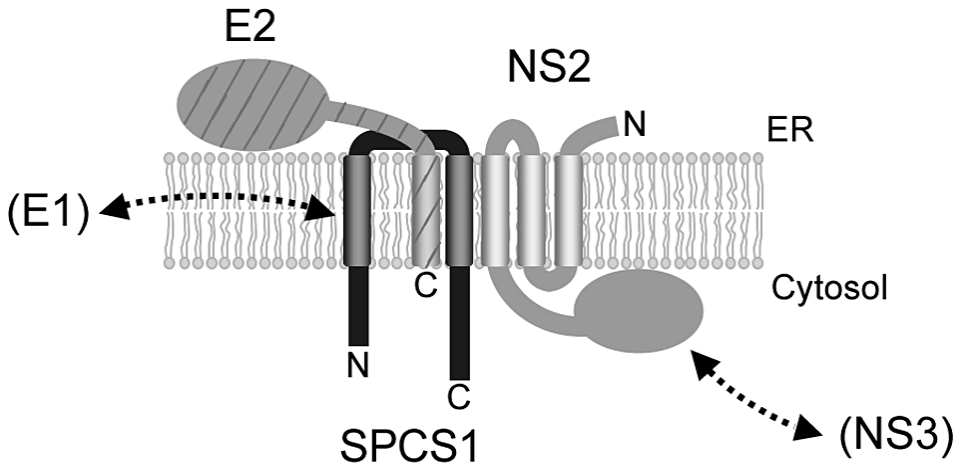
A proposed model for a complex consisting of NS2, SPCS1 and E2 associated with ER membranes.

Further analyses to address the mechanistic implication of SPCS1 on the HCV lifecycle revealed that SPCS1 knockdown impaired the assembly of infectious viruses in the cells, but not cell entry, RNA replication, or release from the cells (Fig. 5). We thus considered the possibility that the SPCS1-NS2 interaction is important for the role of NS2 in viral assembly. Several studies have reported that HCV NS2 is associated biochemically or genetically with viral structural proteins as well as NS proteins [10], [18]–[25]. As an intriguing model, it has been proposed that NS2 functions as a key organizer of HCV assembly and plays a key role in recruiting viral envelope proteins and NS protein(s) such as NS3 to the assembly sites in close proximity to lipid droplets [21]. The interaction of NS2 with E2 has been shown by use of an HCV genome encoding tagged-NS2 protein in virion-producing cells. Furthermore, the selection of an assembly-deficient NS2 mutation located within its TM3 for pseudoreversion leads to a rescue mutation in the TM domain of E2, suggesting an in-membrane interaction between NS2 and E2 [21]. Another study identified two classes of NS2 mutations with defects in virus assembly; one class leads to reduced interaction with NS3, and the other, located in the TM3 domain, maintains its interaction with NS3 but shows impaired interaction between NS2 and E1-E2 [20]. However, the precise details of the NS2-E2 interaction, such as direct protein-protein binding or participating host factors, are unknown. Our results provide evidence that SPCS1 has an important role in the formation of the NS2-E2 complex by its interaction with both NS2 and E2, most likely via their transmembrane domains, including TM3 of NS2. As knockdown of SPCS1 reduced the interaction of NS2 and E2 as shown in Fig. 6D and E, it may be that SPCS1 contributes to NS2-E2 complex formation or to stabilizing the complex. Based on data obtained in this study, we propose a model of the formation of an E2-SPCS1-NS2 complex at the ER membrane (Fig. 7).

In summary, we identified SPCS1 as a novel NS2-binding host factor required for HCV assembly by split-ubiquitin membrane yeast two-hybrid screening. Our data demonstrate that SPCS1 plays a key role in the E2-NS2 interaction via formation of an E2-SPCS1-NS2 complex. These findings provide clues for understanding the molecular mechanism of assembly and formation of infectious HCV particles.

## Materials and Methods

### Split ubiquitin-based yeast two-hybrid screen

A split-ubiquitin membrane yeast two-hybrid screen was performed to identify possible NS2 binding partners. This screening system (DUALmembrane system; Dualsystems Biotech, Schlieren, Switzerland) is based on an adaptation of the ubiquitin-based split protein sensor [26]. The full-length HCV NS2 gene derived from the JFH-1 strain [29] was cloned into pBT3-SUC bait vector to obtain bait protein fused to the C-terminal half of ubiquitin (NS2-Cub) along with a transcription factor. Prey proteins generated from a human liver cDNA library (Dualsystems Biotech) were expressed as a fusion to the N-terminal half of ubiquitin (NubG). Complex formation between NS2-Cub and NubG-protein from the library leads to cleavage at the C-terminus of reconstituted ubiquitin by ubiquitin-specific protease(s) with consequent translocation of the transcription factor into the nucleus. Library plasmids were recovered from positive transformants, followed by determining the nucleotide sequences of inserted cDNAs, which were identified using the BLAST algorithm with the GenBank database.

### Cell culture

Human embryonic kidney 293T cells, and human hepatoma Huh-7 cells and its derivative cell lines Huh7.5.1 [43] and Huh7-25 [36], were maintained in Dulbecco's modified Eagle medium supplemented with nonessential amino acids, 100 U of penicillin/ml, 100 µg of streptomycin/ml, and 10% fetal bovine serum (FBS) at 37°C in a 5% CO_2_ incubator.

### Plasmids

Plasmids pCAGC-NS2/JFH1am and pHHJFH1am were previously described [33]. The plasmid pCAGC-p7/JFHam, having adaptive mutations in E2 (N417S) and p7 (N765D) in pCAG/C-p7 [44], was constructed by oligonucleotide-directed mutagenesis.

To generate the NS2 expression plasmid pCAG F-NS2 and the NS2-deletion mutants, cDNAs encoding the full-length or parts of NS2 possessing the FLAG-tag and spacer sequences (MDYKDDDDKGGGGS) were amplified from pCAGC-NS2/JFH1am by PCR. The resultant fragments were cloned into pCAGGS. For the NS2-NS3 expression plasmid pEF F-NS2-3, a cDNA encoding the entire NS2 and the N-terminal 226 amino acids of NS3 with the N-terminal FLAG-tag sequence as above was amplified by PCR and was inserted into pEF1/myc-His (Invitrogen, Carlsbad, CA). The plasmid pEF F-NS2-3 H956A, having a defective mutation in the protease active site within NS2, was constructed by oligonucleotide-directed mutagenesis.

To generate the NS3 expression plasmid pCAGN-HANS3JFH1, a cDNA encoding NS3 with an HA tag at the N terminus, which was amplified by PCR with pHHJFHam as a template, was inserted downstream of the CAG promoter of pCAGGS.

To generate the SPCS1-expressing plasmid pCAG-SPCS1-myc and its deletion mutants, cDNAs encoding all of or parts of SPCS1 with the Myc tag sequence (EQKLISEEDL) at the C-terminus, which was amplified by PCR, was inserted into pCAGGS. pSilencer-shSPCS1 carrying a shRNA targeted to SPCS1 under the control of the U6 promoter was constructed by cloning the oligonucleotide pair 5′- GATCCGCAATAGTTGGATTTATCTTTCAAGAGAAGATAAATCCAACTATTGCTTTTTTGGAAA-3′ and 5′- AGCTTTTCCAAAAAAGCAATAGTTGGATTTATCTTCTCTTGAAAGATAAATCCAACTATTGCG-3′ between the BamHI and HindIII sites of pSilencer 2.1-U6 hygro (Ambion, Austin, TX). To generate a construct expressing shRNA-resistant SPCS1 pSPCS1-sh^r^, a cDNA fragment coding for SPCS1, in which the 6 bp within the shRNA targeting region (5′-GCAATAGTTGGATTTATCT-3′) was replaced with GCTATTGTCGGCTTCATAT that causes no aa change, was amplified by PCR. The resulting fragment was confirmed by sequencing and then cloned into pCAGGS.

Full-length SPCS1 and N-terminal region of NS2 (aa 1–94) were amplified by PCR and cloned onto EcoRI and HindIII sites of phmKGN-MN and phmKGC-MN, which encode the mKG fragments (CoralHue Fluo-chase Kit; MBL, Nagoya, Japan), designated as pSPCS1-mKG(N) and pNS2-mKG(C), respectively. Transmembrane domain of the E1 to E2 was also amplified by PCR and cloned onto EcoRI and HindIII sites of phmKGC-MN. To avoid the cleavage of E2-mKG(C) fusion protein in the cells, last alanine of the E2 protein was deleted. Positive control plasmids for mKG system, pCONT-1 and pCONT-2, which encode p65 partial domain from NF-κB complex fused to mKG(N) and p50 partial domain from NF-κB complex fused to mKG(C) respectively, were supplied from MBL. For PLA experiments, cDNA for SPCS1 d2-myc with the V5 tag at the N-terminus was amplified by PCR, and inserted into pCAGGS. For expression of HCV E2, cDNA from E1 signal to the last codon of the transmembrane domain of the E2, in which part of the hypervariable region-1 (aa 394–400) were replaced with FLAG-tag and spacer sequences (DYKDDDDKGGG), was amplified by PCR, and inserted into pCAGGS. For expression of FLAG-core, cDNAs encoding Core (aa 1–152) possessing the FLAG-tag and spacer sequences (MDYKDDDDKGGGGS) were amplified from pCAGC191 [45] by PCR. The resultant fragments were cloned into pCAGGS.

### DNA transfection

Monolayers of 293T cells were transfected with plasmid DNA using FuGENE 6 transfection reagent (Roche, Basel, Switzerland) in accordance with the manufacturer's instructions. Huh-7, Huh7.5.1, and Huh7-25 cells were transfected with plasmid DNA using TransIT LT1 transfection reagent (Mirus, Madison, WI).

### PLA

The assay was performed in a humid chamber at 37°C according to the manufacturer's instructions (Olink Bioscience, Uppsala, Sweden). Transfected 293T cells were grown on glass coverslips. Two days after transfection, cells were fixed with 4% paraformaldehyde in phosphate-buffered saline (PBS) for 20 min, then blocked and permeabilized with 0.3% Triton X-100 in a nonfat milk solution (Block Ace; Snow Brand Milk Products Co., Sapporo, Japan) for 60 min at room temperature. Then the samples were incubated with a mixture of mouse anti-FLAG monoclonal antibody M2 and rabbit anti-V5 polyclonal antibody for 60 min, washed three times, and incubated with plus and minus PLA probes. After washing, the ligation mixture containing connector oligonucleotide was added for 30 min. The washing step was repeated, and amplification mixture containing fluorescently labeled DNA probe was added for 100 min. Finally, the samples were washed and mounted with DAPI mounting medium. The signal representing interaction was analyzed by Leica TCS SPE confocal microscope.

### mKG system

The assay was performed according to the manufacturer's instructions (CoralHue Fluo-chase Kit; MBL). 293T cells were transfected by a pair of mKG fusion constructs. Twenty-four hours after transfection, cell were fixed and stained with DAPI. The signal representing interaction was analyzed by Leica TCS SPE confocal microscope.

### Gene silencing by siRNA

The siRNAs were purchased from Sigma-Aldrich (St. Louis, MO) and were introduced into the cells at a final concentration of 10 to 30 nM using Lipofectamine RNAiMAX (Invitrogen). Target sequences of the siRNAs were as follows: SPCS1 #1 (5′-CAGUUCGGGUGGACUGUCU-3′), SPCS1 #2 (5′-GCAAUAGUUGGAUUUAUCU-3′), SPCS1 #3 (5′-GAUGUUUCAGGGAAUUAUU-3′), SPCS1 #4 (5′-GUUAUGGCCGGAUUUGCUU-3′), claudin-1 (5′-CAGUCAAUGCCAGGUACGA-3′), PI4K (5′-GCAAUGUGCUUCGCGAGAA-3′) and scrambled negative control (5′-GCAAGGGAAACCGUGUAAU-3′). Additional control siRNAs for SPCS1 were as follows: C911-#2 (5′-GCAAUAGUaccAUUUAUCU-3′), C911-#3 (5′-GAUGUUUCuccGAAUUAUU-3′) and C911-#4 (5′-GUUAUGGCgccAUUUGCUU-3′). Bases 9 through 11 of the siRNAs replaced with their complements were shown in lower cases.

### Establishment of a stable cell line expressing the shRNA

Huh-7 cells were transfected with pSilencer-SPCS1, and drug-resistant clones were selected by treatment with hygromycin B (Wako, Tokyo, Japan) at a final concentration of 500 µg/ml for 4 weeks.

### Virus

HCVtcp and HCVcc derived from JFH-1 having adaptive mutations in E2 (N417S), p7 (N765D), and NS2 (Q1012R) were generated as described previously [33]. The rAT strain of JEV [46] was used to generate virus stock.

### Antibodies

Mouse monoclonal antibodies against actin (AC-15) and FLAG (M2) were obtained from Sigma-Aldrich (St. Louis, MO). Mouse monoclonal antibodies against flavivirus group antigen (D1-4G2) were obtained from Millipore (Billerica, MA). Rabbit polyclonal antibodies against FLAG and V5 were obtained from Sigma-Aldrich. Rabbit polyclonal antibodies against SPCS1, claudin-1, PI4K and myc were obtained from Proteintech (Chicago, IL), Life Technologies (Carlsbad, CA), Cell Signaling (Danvers, MA) and Santa Cruz Biotechnology (Santa Cruz, CA), respectively. An anti-apoE goat polyclonal antibody was obtained from Millipore. Rabbit polyclonal antibodies against NS2 and NS3 were generated with synthetic peptides as antigens. Mouse monoclonal antibodies against HCV Core (2H9) and E2 (8D10-3) and rabbit polyclonal antibodies against NS5A and JEV are described elsewhere [47].

### Titration

To determine the titers of HCVcc, Huh7.5.1 cells in 96-well plates were incubated with serially-diluted virus samples and then replaced with media containing 10% FBS and 0.8% carboxymethyl cellulose. Following incubation for 72 h, the monolayers were fixed and immunostained with the anti-NS5A antibody, followed by an Alexa Fluor 488-conjugated anti-rabbit secondary antibody (Invitrogen). Stained foci were counted and used to calculate the titers of focus-forming units (FFU)/ml. For intracellular infectivity of HCVcc, the pellets of infected cells were resuspended in culture medium and were lysed by four freeze-thaw cycles. After centrifugation for 5 min at 4,000 rpm, supernatants were collected and used for virus titration as above. For titration of JEV, Huh7.5.1 cells were incubated with serially-diluted virus samples and then replaced with media containing 10% FBS and 0.8% carboxymethyl cellulose. After a 24 h incubation, the monolayers were fixed and immunostained with a mouse monoclonal anti-flavivirus group antibody (D1-4G2), followed by an Alexa Fluor 488-conjugated anti-mouse secondary antibody (Invitrogen).

### Immunoprecipitation

Transfected cells were washed with ice-cold PBS, and suspended in lysis buffer (20 mM Tris-HCl [pH 7.4] containing 135 mM NaCl, 1% TritonX-100, and 10% glycerol) supplemented with 50 mM NaF, 5 mM Na_3_VO_4_, and complete protease inhibitor cocktail, EDTA free (Roche). Cell lysates were sonicated for 10 min and then incubated for 30 min at 4°C, followed by centrifugation at 14,000× *g* for 10 min. The supernatants were immunoprecipitated with anti-Myc-agarose beads (sc-40, Santa Cruz Biotechnology) or anti-FLAG antibody in the presence of Dynabeads Protein G (Invitrogen). The immunocomplexes were precipitated with the beads by centrifugation at 800× *g* for 30 s, or by applying a magnetic field, and then were washed four times with the lysis buffer. The proteins binding to the beads were boiled with SDS sample buffer and then subjected to SDS–polyacrylamide gel electrophoresis (PAGE).

### Immunoblotting

Transfected cells were washed with PBS and lysed with 50 mM Tris-HCl, pH 7.4, 300 mM NaCl, 1% Triton X-100. Lysates were then sonicated for 10 min and added to the same volume of SDS sample buffer. The protein samples were boiled for 10 min, separated by SDS-PAGE, and transferred to polyvinylidene difluoride membranes (Millipore). After blocking, the membranes were probed with the primary antibodies, followed by incubation with peroxidase-conjugated secondary antibody. Antigen-antibody complexes were visualized by an enhanced chemiluminescence detection system (Super Signal West Pico Chemiluminescent Substrate; PIERCE, Rockford, IL) according to the manufacturer's protocol and were detected by an LAS-3000 image analyzer system (Fujifilm, Tokyo, Japan).

### Albumin measurement

To determine the human albumin level secreted from cells, culture supernatants were collected and passed through a 0.45-µm pore filter to remove cellular debris. The amounts of human albumin were quantified using a human albumin ELISA kit (Bethyl Laboratories, Montgomery, TX) according to the manufacturer's protocol.

## Supporting Information

Figure S1 Effects of SPCS1-siRNAs and the C911 mismatch control siRNAs on the expression of SPCS1 and production of HCV. (A) Huh7.5.1 cells were transfected with either siRNAs targeted for SPCS1 (SPCS1-#2, -#3, and -#4), scrambled control siRNA (Scrambled) or C911 siRNA in which bases 9 through 11 of each SPCS1 siRNA were replaced with their complements (C911-#2, -#3, and -#4) at a final concentration of 15 nM, and were infected with HCVcc at a multiplicity of infection (MOI) of 0.05 at 24 h post-transfection. Expression levels of endogenous SPCS1 and actin in the cells were examined by immunoblotting using anti-SPCS1 and anti-actin antibodies at 3 days post-infection. (B) Infectious titers of HCVcc in the supernatant of the infected cells were determined at 3 days postinfection.
(TIF)

Figure S2 293T cells were transfected with E2 expression plasmid in the presence or absence of SPCS1-myc expression plasmid. The cell lysates of the transfected cells were immunoprecipitated with anti-myc antibody. The resulting precipitates and whole cell lysates used in IP were examined by immunoblotting using anti-E2 or anti-myc antibody. An empty plasmid was used as a negative control.
(TIF)

Figure S3 Interaction of HCV E2 with SPCS1 in mammalian cells. (A) 293T cells were transfected with indicated plasmids. 2 days posttransfection, cells were fixed and permeabilized with Triton X-100, then subjected to in situ PLA (Upper) or immunofluorescence staining (Lower) using anti-FLAG and anti-V5 antibodies. (B) Detection of the SPCS1-E2 interaction in transfected cells using the mKG system. 293T cells were transfected by indicated pair of mKG fusion constructs. Twenty-four hours after transfection, cell were fixed and stained with DAPI, and observed under a confocal microscope.
(TIF)
