# Supplementary figures and images for: Signal Peptidase Complex Subunit 1 Participates in the Assembly of Hepatitis C Virus through an Interaction with E2 and NS2
Source: PLoS Pathog. 2013 Aug 29;9(8):e1003589. doi: 10.1371/journal.ppat.1003589 (PMC3757040; doi:10.1371/journal.ppat.1003589)

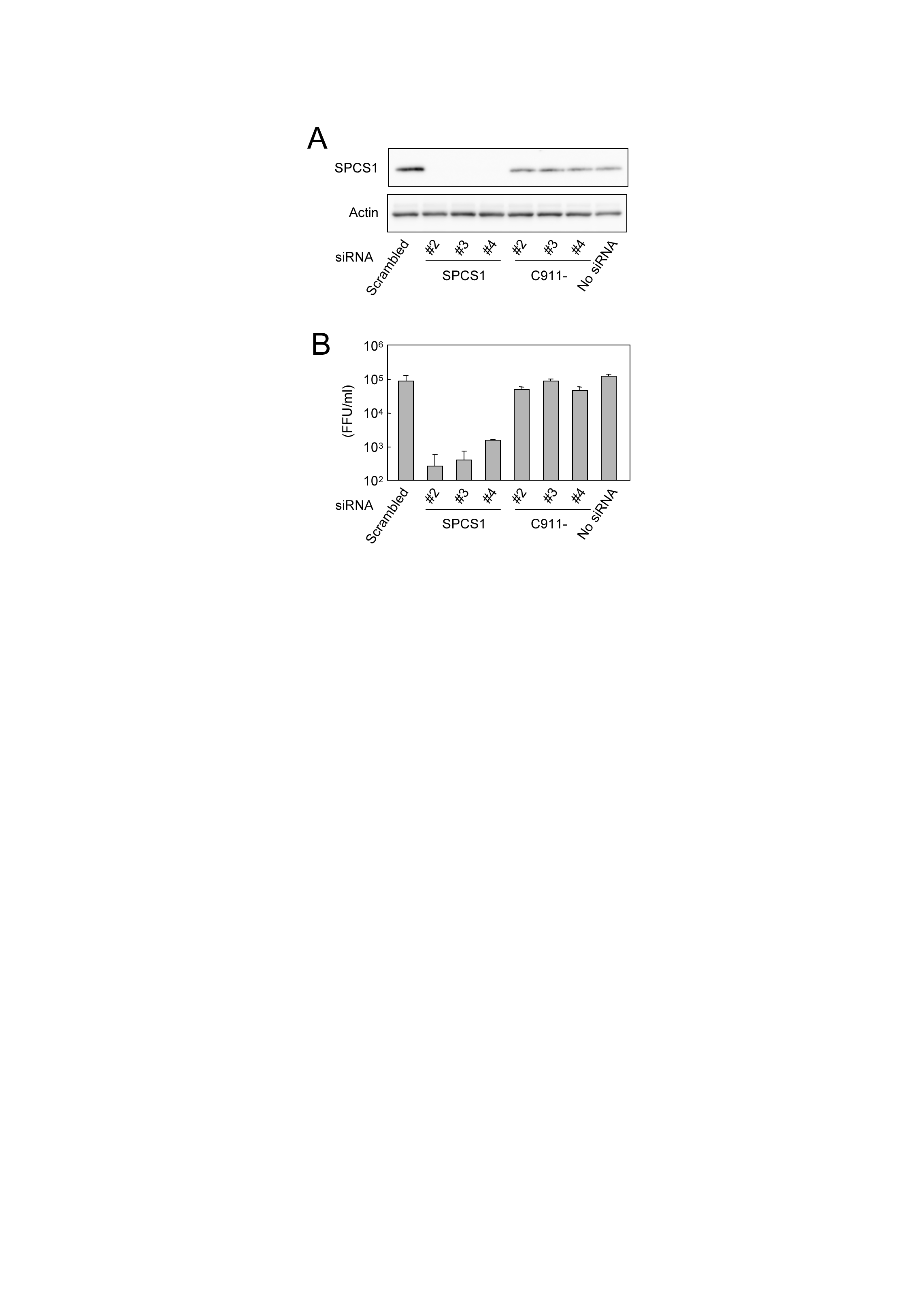

Supplement: Figure S1 — Effects of SPCS1-siRNAs and the C911 mismatch control siRNAs on the expression of SPCS1 and production of HCV. (A) Huh7.5.1 cells were transfected with either siRNAs targeted for SPCS1 (SPCS1-#2, -#3, and -#4), scrambled control siRNA (Scrambled) or C911 siRNA in which bases 9 through 11 of each SPCS1 siRNA were replaced with their complements (C911-#2, -#3, and -#4) at a final concentration of 15 nM, and were infected with HCVcc at a multiplicity of infection (MOI) of 0.05 at 24 h post-transfection. Expression levels of endogenous SPCS1 and actin in the cells were examined by immunoblotting using anti-SPCS1 and anti-actin antibodies at 3 days post-infection. (B) Infectious titers of HCVcc in the supernatant of the infected cells were determined at 3 days postinfection. (TIF) [file ppat.1003589.s001.tif]

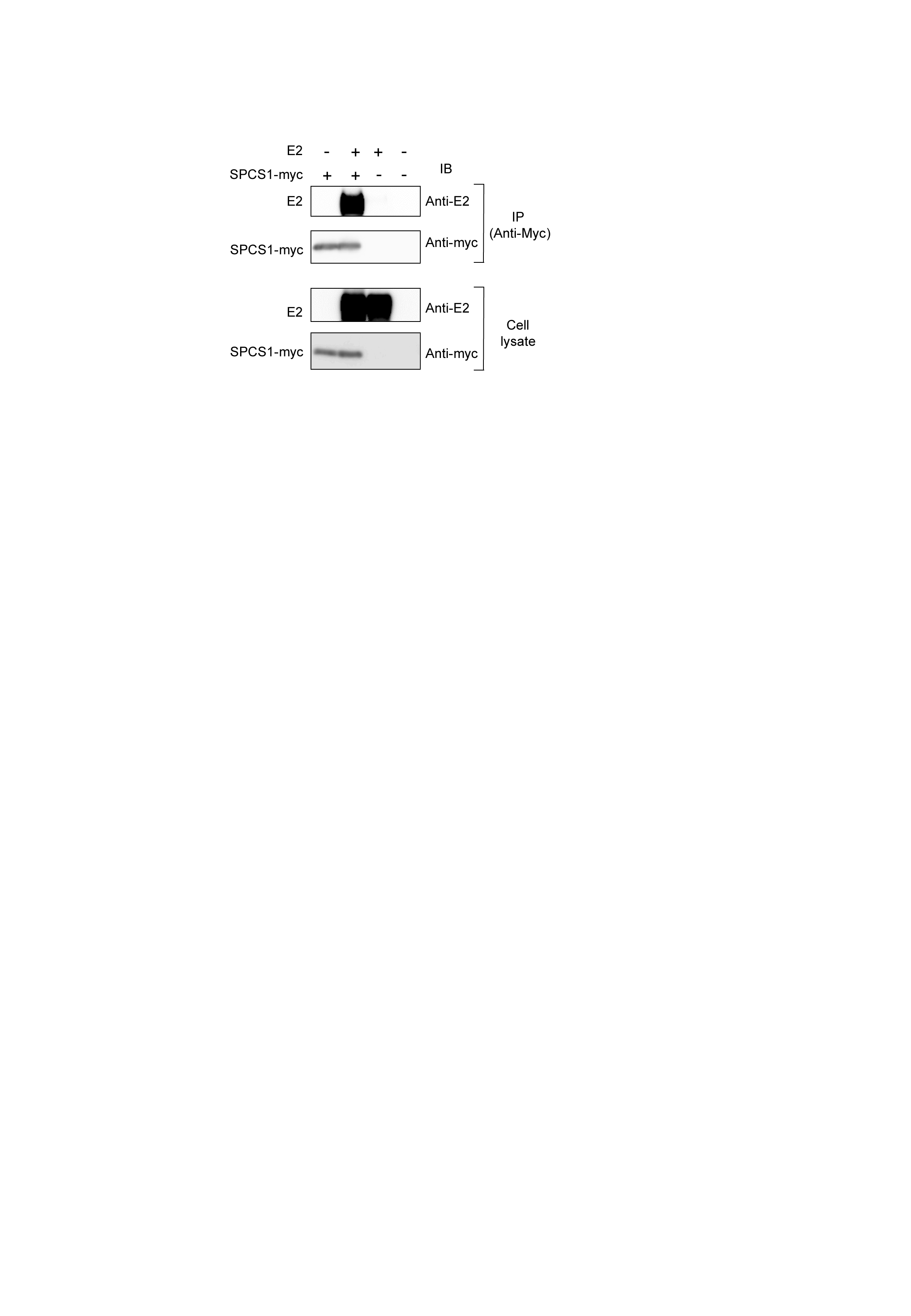

Supplement: Figure S2 — 293T cells were transfected with E2 expression plasmid in the presence or absence of SPCS1-myc expression plasmid. The cell lysates of the transfected cells were immunoprecipitated with anti-myc antibody. The resulting precipitates and whole cell lysates used in IP were examined by immunoblotting using anti-E2 or anti-myc antibody. An empty plasmid was used as a negative control. (TIF) [file ppat.1003589.s002.tif]

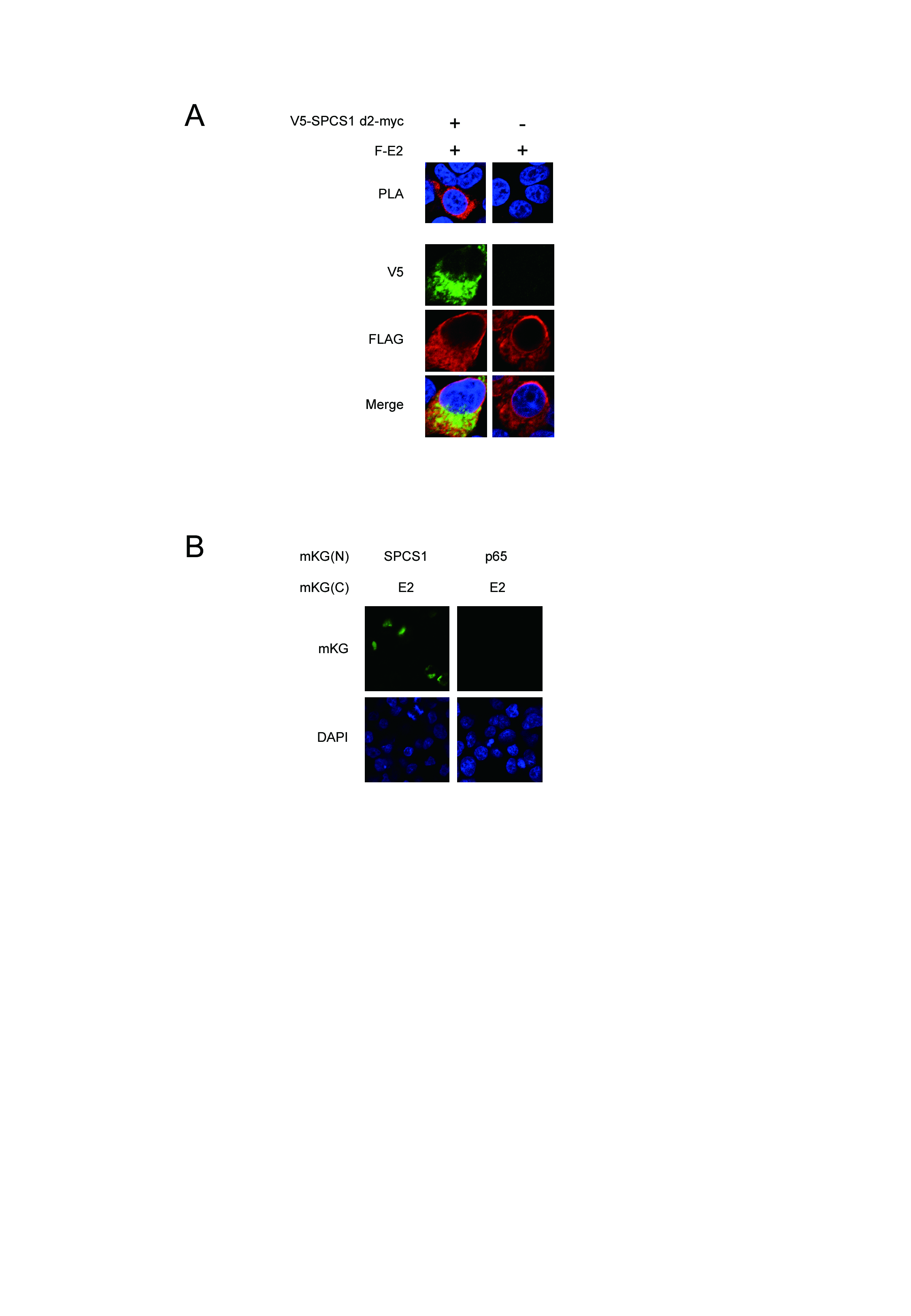

Supplement: Figure S3 — Interaction of HCV E2 with SPCS1 in mammalian cells. (A) 293T cells were transfected with indicated plasmids. 2 days posttransfection, cells were fixed and permeabilized with Triton X-100, then subjected to in situ PLA (Upper) or immunofluorescence staining (Lower) using anti-FLAG and anti-V5 antibodies. (B) Detection of the SPCS1-E2 interaction in transfected cells using the mKG system. 293T cells were transfected by indicated pair of mKG fusion constructs. Twenty-four hours after transfection, cell were fixed and stained with DAPI, and observed under a confocal microscope. (TIF) [file ppat.1003589.s003.tif]
